# Patterns of Body Composition Changes and Their Predictors During Cancer Therapy in Newly Diagnosed Adult Oncology Patients With Varying Baseline Nutritional Status at a Tertiary Hospital in Ethiopia: A Prospective Cohort Study

**DOI:** 10.1002/cnr2.70617

**Published:** 2026-07-02

**Authors:** Abebe Dukessa Dubiwak, Mulualem Tadesse, Tefera Belachew, Henok Gulilat, Belay Zawdie, Selam Tesfaye, Yohannes Tesfaye, Melesa Sinaga Teshome, Lelisa Sena Dadi

**Affiliations:** ^1^ Department of Epidemiology and Biostatistics, Faculty of Public Health Jimma University Jimma Ethiopia; ^2^ Department of Biomedical Sciences, Faculty of Medical Sciences Jimma University Jimma Ethiopia; ^3^ School of Medical Laboratory Sciences, Faculty of Health Sciences Jimma University Jimma Ethiopia; ^4^ Department of Microbiology and Parasitology College of Medicine and Health Sciences, University of Rwanda Huye Rwanda; ^5^ Department of Nutrition and Dietetics, Faculty of Public Health Jimma University Jimma Ethiopia; ^6^ Department of Clinical Oncology, Faculty of Medical Sciences Jimma University Jimma Ethiopia

**Keywords:** body composition, cancer, malnourished, pattern, well‐nourished

## Abstract

**Background:**

Cancer incidence is an emerging public health problem in low‐ and middle‐income countries. Despite strong recommendations from the European Society for Clinical Nutrition and Metabolism expert to expand nutritional status assessment practices including body composition analysis for newly diagnosed cancer patients, resource‐limited settings like Ethiopia have yet to implement body composition assessments.

**Aims:**

This study aimed to demonstrate change in various body composition parameters, their patterns, and predictors among newly diagnosed cancer patients.

**Methods:**

A prospective cohort study design was applied on 231 newly diagnosed adult cancer patients at the oncology department of Jimma University Medical Center between 2024 and 2025. Analysis of body composition was done three times starting from prior to chemotherapy initiation (baseline) at about 3 months intervals using bioelectric impedance analyzer. Chi‐squared and independent *t*‐test statistical models were used to assess the variation of body composition between well‐nourished and malnourished, while paired *t*‐test was used to assess change prior to and post treatment, and linear mixed model was used to identify predictors of patient body composition.

**Results:**

There were significant differences in body composition parameters between well‐nourished and malnourished cancer patients. A decreasing pattern in almost all body composition parameters except fat mass was observed in malnourished cancer patients. Aging (*β* = −0.033; 95% CI: −0.054, −0.013), advanced cancer stages (*β* = −2.49; 95% CI: −3.90, −1.08), and male sex (*β* = 1.82, 95% CI: 1.26, 2.37) were found to be predictors of SMMI trajectories among the cancer patients.

**Conclusions:**

During chemotherapy, malnourished patients experienced a decline of body composition parameters. Thus, monitoring body weight alone can be misleading, as fat mass gain masks change in the overall nutritional status. Therefore, considering early nutritional assessment, body composition analysis in the diagnosis of cancer, can benefit the patients and mitigate potential complications.

## 1 Introduction

Cancer incidence has been rising significantly worldwide. There were an estimated 20 million new cancer cases and 9.7 million deaths across the globe in 2022 alone [1]. This public health problem is especially concerning in low‐ and middle‐income countries (LMICs), where 75% of global deaths attributed to cancer now occur [2]. Malnutrition among cancer patients is widespread since they are at a higher risk for developing it [3, 4]. Cancer patients' prognosis is significantly worsened by malnutrition [5]. Besides cachexia, poor nutritional status is a universally recognized negative prognostic factor and significantly reduces the efficacy of treatment and overall chances of survival in cancer patients [6, 7]. Thus, more than 20% of cancer‐related deaths are attributed to cancer‐associated malnutrition [8, 9].

Cancer‐associated malnutrition differs from starvation‐related malnutrition because it is a result of multiple and interrelated mechanisms and pathways [3, 6]. Newly diagnosed cancer patients often face psychological distress, treatment side effects, and disease burden, leading to reduced physical function, appetite loss, and tissue degradation [10, 11, 12, 13]. The mixed effect of appetite loss and degradation of tissue's biomolecules lead to significant loss of body weight and alterations in body composition [14].

Nowadays, malnutrition screening tools often rely on the overall body weight change, which results in an insufficient and delayed diagnosis [8]. However, cancer patients experience not only loss of overall body weight but also loss of muscle tissue and body cell mass (BCM) [15], and changes in fluid distribution with extracellular expansion and reduced intracellular water [16, 17]. Thus, even though progressive weight loss is a typical hallmark of many cancer types [5], managing patients' nutritional needs based only on body weight can be misleading, as it does not reflect body composition [16, 17].

Nevertheless, body composition is demonstrated to be instructive in clinical nutrition assessment because it precisely distinguishes body components into categories, such as muscle tissue, adipose tissue, and bone [5]. Therefore, ascertainment of body composition change, such as the loss of fat‐free mass, is thought to be more powerful than weight loss in determining the nutritional status of patients [14].

Additionally, the body composition assessment using bioelectric impedance analyzer (BIA) output parameters such as phase angle (PA), fat mass (FM), skeletal muscle mass (SMM), and fat‐free mass (FFM) has been broadly studied as predictors of nutritional status and survival in cancer patients [17, 18, 19, 20, 21, 22, 23]; hence, body composition measures have received increasing attention as potential indicators of the real nutritional status of the cancer patients [24].

Understanding of body composition change among cancer patients receiving treatment can aid oncologists in predicting clinical outcomes and prognosis, as body composition precisely reflects a patient's nutritional status [25]. However, few published studies have examined the utility of body composition obtained from BIA to monitor the effects of cancer therapy on cancer patients [26, 27]. To the best of our knowledge, no studies have examined the patterns of body composition change among newly diagnosed cancer patients during chemotherapy course in low‐ and middle‐income countries (LMICs), particularly in Ethiopia.

Therefore, conducting repeated nutritional assessment by measuring body composition would help early detection of malnutrition related complications. Subsequently, this approach plays a crucial role in effective management of cancer‐associated malnutrition, ultimately increasing cancer patients' survival and can reduce medical/healthcare costs [4, 28, 29, 30, 31]. Thus, this study aimed to ascertain the various body composition parameters changes and their patterns throughout the course of therapy and its predictors among newly diagnosed cancer patients during chemotherapy with different baseline nutritional statuses undergoing cancer therapy at a Tertiary Hospital of Ethiopia.

## 2 Methods and Materials

### 2.1 Study Design and Enrollment Criteria

A prospective cohort study was conducted involving 231 newly diagnosed adult oncology patients. Enrollment occurred between June 2024 and December 2024, with follow‐up assessments carried out from June 2024 to June 2025 at the Cancer Treatment Center (CTC) of Jimma University Medical Center (JUMC). The patients were followed from the time of histopathologically confirmed diagnosis of cancer until the end of chemotherapy treatment. Newly histopathologically confirmed adult cancer patients (≥ 18 years) who had not yet initiated chemotherapy but were scheduled to receive chemotherapy or concurrent chemo‐radiotherapy were included in this study. Patients were excluded if their chemotherapy plan involved fewer than three cycles or a single‐shot regimen; if they had a history of previous cancer diagnosis or recurrence; if they were not scheduled for chemotherapy; or if they had conditions that interfered with body composition assessment, such as the presence of a metal prosthesis or pacemaker, limb amputation, being bedridden, or severe extremity edema. Patients planned for fewer than three chemotherapy cycles were excluded to minimize loss to follow‐up, as repeated measurements conducted over a six‐month period. These patients typically lacked regular follow‐up appointments, rendering longitudinal assessments impracticable. The cancer staging system employed in this study was based on the tumor–node–metastasis (TNM) classification.

### 2.2 Sample Size and Sampling Procedures

Since this study was more than one follow‐up measurement and the purpose of the study was to estimate the change over time, the sample size determination for the follow‐up study developed by Twisk JWR was used [32]. So the final sample size needed was estimated to be 231 considering certain differences in a continuous outcome variable statistically significant at the 5% level of confidence and power of 80%; within‐subject correlation coefficients (*ρ* = 0.25), expected standard deviation units difference = 0.2, three follow‐up measurements (T), and adding a 10% dropout rate. Consecutive sampling procedure was used to recruit the study participants.

### 2.3 Data Collection Instruments and Procedures

**FIGURE 1:**
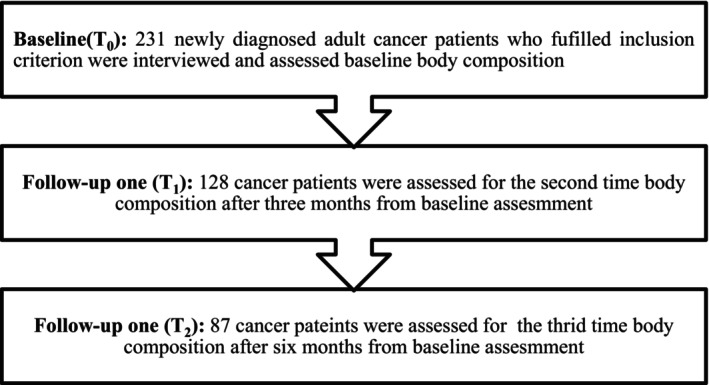
Schematic representation of number of study samples in each body composition assessment at Jimma Medical Center, 2025.

The data collection tools (DCTs) were developed on KOBOTOOLBOX. The DCTs comprise sociodemographic characteristics, behavioral factors, medical, and Eastern Cooperative Oncology Group (ECOG) performance status scale, BIA parameters and inflammatory biomarkers. Patients' body composition assessment was carried out three times at three‐month intervals starting from baseline after confirming diagnosis of cancer but before initiation of cancer treatments (T_0_), after nearly completing 50% of planned treatment (T_1_), and after completing the planned treatment (T_2_) (Figure 1). Chemotherapy is known to significantly affect both body composition and the nutritional status of patients. To minimize potential confounding bias, we defined baseline strictly as the period following cancer diagnosis but prior to the initiation of chemotherapy. This ensured that all measurements reflected patients' nutritional and body composition status before exposure to treatment effects. Therefore, body composition was assessed at 3 and 6 months from baseline to capture both early treatment‐related alterations and medium‐term trajectories. These assessment intervals were selected to align with standard oncology follow‐up schedules, thereby ensuring clinical relevance while maintaining methodological rigor. Moreover, the 6‐month time point encompassed the full course of chemotherapy, as all patients were able to complete treatment within this period. Body composition analysis was performed using a BIA machine (Bodystat 1500 MDD, Richmond Scientific, UK) after fasting at least 8 h, an empty bladder, with the patient lying supine on a bed with legs apart and arms not touching the trunk. The four standard electrodes were positioned on the ulnar aspect of the right wrist and the right medial malleolus, according to the standardized technique [33, 34]. The BIA input variables were age, sex, weight, and height; afterward, the following complete body composition analysis displays on the device FM, lean body mass (LBM), dry lean weight (DLW), BCM, fat‐free mass index (FFMI), total body water (TBW), impedance, resistance (RZ), reactance (XC), PA. Other information was also provided, including metabolic rates, body mass index (BMI), intra‐ and extracellular water (ICW and ECW). SMM was calculated using Janssen et al. [35] equation which is depicted as follows:

(1) $$\begin{array}{cc} \mathrm{SMM}\;\left(\mathrm{kg}\right)= & \;\left[\left({\mathrm{Ht}}^{2}/\mathrm{BIA}\;\text{resistance}\times 0.401\right)\right.\\ & \;\left.+\left(\text{Gender}\times 3.825\right)–\left(\mathrm{age}\times 0.071\right)\right]+5.102 \end{array}$$

where SMM; skeletal muscle mass, Ht; height in centimeters, BIA resistance; Bioelectric impedance analysis resistance in ohms, for gender, male = 1 and women = 0 and age is in years. This equation is used to determine SMM from BIA resistance, which was developed and cross‐validated against magnetic resonance measurements of whole‐body muscle mass. Absolute muscle mass (kg) normalized for squared height using the formula muscle mass (kg)/height (m)^2^ which is called skeletal muscle mass index (SMI) [36]. Anthropometric data, including body weight (usual and current) and height, were measured using a digital scale with a stadiometer (Victoria DX). The BMI was determined using weight in kg divided by height squared in meters and the percentage of weight loss was calculated as usual weight minus current weight divided by usual weight times 100. The patients' functional status was evaluated using the Eastern Cooperative Oncology Group (ECOG) Performance Status Scale. Patients' baseline nutritional status was assessed using the global leadership initiative on malnutrition (GLIM) criteria [37]. The GLIM framework comprises five core criteria, grouped into phenotypic and etiologic categories. Phenotypic criteria include unintentional weight loss, low body mass index, and reduced muscle mass, while etiologic criteria encompass reduced food intake or assimilation, and presence of inflammation. A diagnosis of malnutrition requires the presence of at least one phenotypic criterion in combination with one etiologic criterion [38, 39]. After completing a clinical data collection, five milliliters of blood were drawn from every study participant for serum albumin and C‐reactive protein (CRP) using the Cobas 6000 (Germany) clinical chemistry analyzer. Albumin and CRP were used to calculate modified Glasgow prognostic score (mGPS), while CRP specifically contributes to inflammation assessment for etiologic criteria of GLIM. Body composition parameters were treated as outcome variables, while age, sex, duration of illness, cancer stage, performance status, and co‐morbidities were considered explanatory variables. Co‐morbidities are defined as non‐cancer health conditions that coexist with a cancer diagnosis [40]. In this study, hypertension, diabetes mellitus, other cardiovascular diseases, chronic kidney disease, and chronic respiratory diseases were considered as co‐morbidities.

### 2.4 Data Analysis Procedures

The collected data were exported to R statistical software (version 4.3.3) for cleaning, recoding, exploration, and analysis. Baseline clinical‐demographic characteristics were assessed using Chi ‐square, and Fisher Exact Test for categorical variables, and independent *t*‐test for continues variables. To evaluate changes in body composition before and after cancer therapy, paired *t*‐test was applied. The predictors of longitudinal trajectories of body composition parameters were identified using stratified linear mixed effect model (LMM) after data being converted from wide to long form. In this study, predictors were identified for mean change overtime (trajectories) of SMMI, FFMI, and BFMI as outcome variables, since those variables have many clinical implications [41, 42]. Prior to LMM analysis, descriptive statistics and profile plots, including individual trajectories, mean profiles, and variance plots were examined to characterize the overall structure and distribution of the data. Individual profiles provided insight into within and between subjects variability, while mean profiles were used to assess linearity of change over time. Model assumptions for respective models were checked to ensure validity. Missing data were assumed to be Missing At Random (MAR). To assess robustness, sensitivity analyses were conducted under Missing Not At Random (MNAR) assumption using three approaches regarding missing data: (1) a linear mixed‐effects model, which accommodates missing repeated outcome measures by utilizing all available time points without case deletion or imputation; (2) multiple imputation procedures; and (3) a complete‐case analysis excluding participants with missing measurements. Comparative evaluation demonstrated that the first model yielded lower Akaike Information Criterion (AIC) and Bayesian Information Criterion (BIC) values, indicating a superior model fit. Accordingly, the results presented herein are derived from the LMM without imputation or case removal. Variable selection for the multivariable model was performed using stepwise procedures guided by AIC and BIC values. Predictors rationed in the final model with *p* < 0.05 were considered statistically significant.

## 3 Results

### 3.1 Sociodemographic and Clinical Characteristics of Study Participants

At baseline, 231 participants were enrolled in the study. Of these, 128 participants completed the second measurement, and 87 participants completed the third measurement (Figure 1). Significant differences were observed in several baseline characteristics including nutritional status, weight, BMI, fat‐free mass index (FFMI), and skeletal muscle mass index (SMMI) between participants who completed follow‐up assessments and those who did not (Table 1).

**TABLE 1:** Baseline characteristics of participants: missed vs. completed follow‐up at Jimma Medical Center, 2025.

Baseline characteristics	Completed follow‐up (*N* = 87)	Missed follow‐up (*N* = 144)	Mean difference (95% CI)	*p*
Age (Mean ± SD)	44.36 ± 12.81	47.83 ± 13.95	−3.48 (−7.10, 0.15)	0.06
Nutritional status (*N*)				
Well‐nourished	57 (65.52%)	59 (40.97%)		**< 0.001**
Malnourished	30 (34.48)	85 (59.03)		
Weight (Mean ± SD)	57.68 ± 11.88	52.00 ± 10.23	5.67 (2.76, 8.59)	**< 0.001**
BMI (Mean ± SD)	21.97 ± 4.57	20.04 ± 3.65	1.93 (0.79, 3.07)	**0.001**
Advance stageAdvance stage				
No (stage‐I&II)	15 (17.24%)	27 (18.75%)		0.77
Yes(stage‐III&IV)	72 (82.76)	117 (81.25%)		
Co‐morbidities				
No	74 (85.06%)	130 (90.28%)		0.23
Yes	13 (14.94%)	14 (9.72%)		
LBM (Mean ± SD)	37.15 ± 7.48	33.59 ± 9.56	3.56 (1.33, 5.78)	**0.002**
BFMI (Mean ± SD)	7.43 ± 2.83	7.03 ± 2.48	0.40 (−0.29, 1.10)	0.26
FFMI (Mean ± SD)	14.16 ± 2.55	13.31 ± 2.27	0.85 (0.21, 1.48)	**0.01**
SMMI (Mean ± SD)	6.79 ± 1.26	6.42 ± 1.26	0.37 (0.04, 0.71)	**0.03**

*Note:* Continuous variables were compared using independent samples *t*‐tests, and categorical variables using Chi‐squared or Fisher's exact test as appropriate. *p*‐value < 0.05 (In bold).

Abbreviations: BFMI, body fat mass index; BMI, body mass index; FFMI, fat‐free mass index; LBM, lean body mass; SD, standard deviation; SMMI, skeletal muscle mass index.

From the 231 participants, 71.0% were female, and 52.8% of them were from rural settings. The mean age and BMI of the study participants were 46.52 years and 20.85 kg/m^2^, respectively. More than one‐fourth (28.6%) of them were diagnosed with gynecologic cancer type, more than half (54.1%) of them were at stage IV and palliative treatment intent, and the majority of the study participants (88.3%) had no co‐morbidity. Two‐thirds (66.7%) of the study participants received chemotherapy and the mean duration of illness of the study participants was 10.16 months. More than two‐thirds (69.7%) of study participants' modified Glasgow prognostic score (mGPS) was two, and nearly two‐fifths (38.1%) of them were classified under 1 ECOG performance status, which means they were unable to do strenuous activities but were able to carry out light housework and sedentary activities. Baseline measurement of body compositions such as mean BMI, LBM, DLW, SMMI, fat‐free mass index (FFMI), body fat mass index (BFMI), BCM and PA were significantly lower in malnourished than the corresponding levels in well‐nourished (Table 2).

**TABLE 2:** Baseline clinical‐demographic characteristics of study participants, Jimma Medical Center, 2025.

Variables	Category	Overall (*N* = 231)	Well‐Nourished (*N* = 116)	Malnourished (*N* = 115)	*p*
Age	Mean ± SD	46.52 ± 13.61	44.28 ± 13.17	48.78 ± 13.73	**0.012**
Sex	Female	164 (71.0%)	83 (71.6%)	81 (70.4%)	0.852
	Male	67 (29.0%)	33 (28.4%)	34 (29.6%)	
Residence	Urban	109 (47.2%)	60 (55.0%)	49 (45.0%)	0.165
	Rural	122 (52.8%)	56 (45.9%)	66 (54.1%)	
Weight	Mean ± SD	54.14 ± 11.20	58.60 ± 11.34	49.64 ± 9.09	**< 0.001**
Height	Mean ± SD	161.10 ± 9.19	161.08 ± 9.39	161.12 ± 9.02	0.972
Body composition parameters	BMI	20.85 ± 3.92	22.62 ± 4.10	19.06 ± 2.76	**< 0.001**
	LBM	34.93 ± 8.70	39.94 ± 8.33	32.91 ± 9.21	**< 0.001**
	DLW	8.44 ± 4.69	9.21 ± 3.86	7.66 ± 5.32	**0.012**
	SMMI	6.56 ± 1.27	6.79 ± 1.25	6.33 ± 1.26	**0.005**
	FFMI	13.63 ± 2.41	14.34 ± 2.60	12.92 ± 1.98	**< 0.001**
	BFMI	7.18 ± 2.62	7.80 ± 2.99	6.56 ± 2.01	**< 0.001**
	BCM	21.26 ± 5.36	22.52 ± 5.32	19.99 ± 5.11	**< 0.001**
	PA	5.92 ± 3.05	6.42 ± 2.01	5.41 ± 1.90	**0.012**
	ECW/ICW ratio	0.67 ± 0.13	0.68 ± 0.20	0.65 ± 0.15	0.837
Type of cancer	Breast	54 (23.4%)	35 (30.2%)	19 (16.5%)	0.121
	Gynecologic	66 (28.6%)	30 (25.9%)	36 (31.3%)	
	GIT	58 (25.1%)	29 (25.0%)	29 (25.2%)	
	Head and Neck	21 (9.1%)	9 (7.8%)	12 (10.4%)	
	Hematology, bone and soft tissue	20 (8.7%)	10 (8.6%)	10 (8.7%)	
	Others*	12 (5.1%)	3 (2.5%)	9 (7.9%)	
Stage of cancer	Stage‐I	8 (3.5%)	4 (3.5%)	4 (3.5%)	**0.016**
	Stage‐II	34 (14.7%)	20 (17.2%)	14 (12.2%)	
	Stage‐III	64 (27.7%)	41 (35.3%)	23 (20.0%)	
	Stage‐IV	125 (54.1%)	51 (44.0%)	74 (64.3%)	
Duration of illness	Mean ± SD	10.16 ± 6.49	9.05 ± 4.57	11.27 ± 7.84	**0.009**
Type of treatment	Chemotherapy	154 (66.7%)	78 (50.6%)	76 (49.4%)	0.89
	Chemo‐radiotherapy	77 (33.3%)	38 (49.4%)	39 (50.6%)	
Intent of treatment	Curative	106 (45.9%)	67 (57.8%)	39 (33.9%)	**< 0.001**
	Palliative	125 (54.1%)	49 (42.2%)	76 (66.1%)	
Co‐morbidity status	No	204 (88.3%)	103 (50.5%)	101 (49.5%)	0.819
	Yes	27 (11.7%)	13 (48.1%)	14 (51.9%)	
mGPS	0	24 (10.4%)	13 (54.3%)	11 (45.8%)	0.717
	1	46 (19.9%)	25 (54.3%)	21 (45.7%)	
	2	161 (69.7%)	78 (48.4%)	83 (51.6%)	
Performance Status	ECOG 0	81 (35.1%)	44 (37.9%)	37 (32.2%)	0.189
	ECOG 1	88 (38.1%)	47 (40.5%)	41 (35.6%)	
	ECOG ≥ 2	62 (26.8%)	25 (21.6%)	37 (32.2%)	

*Note:* Continuous variables were compared using independent samples *t*‐tests, and categorical variables using Chi‐squared or Fisher's exact tests as appropriate. *p*‐value < 0.05 (In bold).

Abbreviations: BCM, body cell mass; BFMI, body fat mass index; BMI, body mass index; CRP, C‐reactive protein; DLW, dry lean weight; ECOG, Eastern Cooperative Oncology Group; ECW, extracellular water; FFMI, fat‐free mass index; GIT, gastro‐intestinal tract; ICW, intracellular water; LBM, lean body mass; mGPS, Modified Glasgow Prognostic Score; PA, phase angle; SMMI, skeletal muscle mass index; TBW, total body water.

### 3.2 Comparison of Body Composition Parameters Between Well‐Nourished and Malnourished at Post Cancer Therapy

This study demonstrated statistically significant differences between well‐nourished and malnourished cancer patients at 6 months post‐therapy (relative to baseline) with respect to body weight, BMI, dry lean mass (DLW), SMMI, and fat‐free mass index (FFMI). The mean values of weight, BMI, DLW, SMMI, and FFMI were consistently higher in well‐nourished patients, with mean differences approximately 10 kg, 3 kg/m^2^, 3 kg, 1 kg/m^2^ and 3 kg/m^2^, respectively. Although not statistically significant, other body composition parameters including LBM, BFMI, BCM, PA, and extracellular water to intracellular water (ECW/ICW) ratio also tended to be higher among well‐nourished patients compared with malnourished patients at 6 months post‐therapy (Table 3).

**TABLE 3:** Comparison of body composition between well‐nourished and malnourished at post cancer therapy at JUMC, 2025.

Body composition parameters	Well‐Nourished (*N* = 57)	Malnourished (*N* = 30)	Mean Difference (95% CI)	*p*
Weight	61.42 ± 11.98	51.57 ± 8.55	9.85 (4.95, 14.76)	**< 0.001**
BMI	23.31 ± 4.61	19.99 ± 2.80	3.31 (1.48, 5.14)	**0.001**
LBM	34.55 ± 9.02	31.84 ± 8.74	2.72 (−1.29, 6.70)	0.181
DLW	10.06 ± 4.61	7.39 ± 3.69	2.67 (0.73, 4.61)	**0.008**
SMMI	6.92 ± 1.10	6.18 ± 1.32	0.74 (0.21, 1.27)	**0.007**
FFMI	15.13 ± 1.53	12.64 ± 1.69	2.49 (1.78, 3.20)	**< 0.001**
BFMI	9.08 ± 2.91	8.87 ± 2.87	0.21 (−1.09, 1.51)	0.747
BCM	21.76 ± 5.37	20.41 ± 5.24	1.35 (−1.04, 3.73)	0.266
PA	8.34 ± 6.39	7.66 ± 4.19	0.68 (−1.89, 3.25)	0.600
ECW/ICW ratio	0.63 ± 0.14	0.61 ± 0.15	0.02 (−0.04, 0.09)	0.498

*Note:* Continuous variables were compared using independent samples *t*‐tests. *p*‐value < 0.05 (In bold).

Abbreviations: BCM, body cell mass; BFMI, body fat mass index; BMI, body mass index; ECW, extracellular water; FFMI, fat‐free mass index; ICW, intracellular water; LBM, lean body mass; PA, phase angle; SMMI, skeletal muscle mass index; TBW, total body water.

### 3.3 Change of Body Composition Parameters Before and After Cancer Therapy

This study pointed out that at post‐cancer therapy there were statistically significant decrements of mean LBM (Mean difference (MD) = −4.88, 95% CI: −7.58, −2.17) and ECW/ICW ratio (MD = −0.07, 95% CI: −0.12, −0.03) among well‐nourished cancer patients. However, mean BFMI (MD = 1.33, 95% CI: 0.30, 2.35) and PA (MD = 2.06, 95% CI: 0.28, 3.85) showed statistically significant increments after post‐cancer therapy. On the other hand, only mean BFMI showed statistically significant increment among the malnourished cancer patients (MD = 2.05, 95% CI: 0.77, 3.32). Other body composition such as mean SMMI and FFMI showed increments in well‐nourished and decrements in malnourished cancer patients numerically though statistically insignificant (Table 4).

**TABLE 4:** The variation in body composition prior and post cancer therapy in well‐nourished and malnourished at JUMC, 2025.

Body composition parameters	Well‐nourished (*N* = 57)				Malnourished (*N* = 30)			
	Prior to cancer therapy	Post cancer therapy	Mean difference (95% CI)	*p*	Prior to cancer therapy	Post cancer therapy	Mean difference (95% CI)	*p*
Weight	61.82 ± 11.28	61.42 ± 11.98	−0.40 (−1.69, 0.89)	0.534	49.80 ± 8.66	51.57 ± 8.55	+1.77 (−0.52, 4.05)	0.124
BMI	23.55 ± 4.57	23.31 ± 4.61	−0.25 (−0.89, 0.40)	0.448	18.95 ± 2.70	19.99 ± 2.80	+1.04 (−0.08, 2.16)	0.066
LBM	39.43 ± 6.30	34.55 ± 9.02	−4.88 (−7.58, −2.17)	**0.001**	32.81 ± 7.73	31.84 ± 8.74	−0.98 (−4.69, 2.73)	0.594
DLW	10.11 ± 3.20	10.06 ± 4.61	−0.05 (−1.50, 1.39)	0.942	6.32 ± 3.81	7.39 ± 3.69	+1.06 (−0.82, 2.95)	0.258
SMMI	6.86 ± 1.13	6.92 ± 1.10	+0.06 (−0.23, 0.37)	0.663	6.67 ± 1.49	6.18 ± 1.32	−0.48 (−1.13, 0.17)	0.138
FFMI	14.53 ± 2.77	15.13 ± 1.53	+0.60 (−0.25, 1.44)	0.162	13.44 ± 1.90	12.64 ± 1.69	−0.80 (−1.69, 0.08)	0.073
BFMI	7.75 ± 3.14	9.08 ± 2.91	+1.33 (0.30, 2.35)	**0.012**	6.82 ± 2.03	8.87 ± 2.87	+2.05 (0.77, 3.32)	**0.003**
BCM	23.32 ± 4.74	21.76 ± 5.37	−1.57 (−3.29, 0.16)	0.074	20.28 ± 6.22	20.41 ± 5.24	+0.13 (−3.10, 3–37)	0.935
PA	6.28 ± 2.19	8.34 ± 6.39	+2.06 (0.28, 3.85)	**0.024**	6.32 ± 2.47	7.66 ± 4.19	+1.34 (−0.49, 3.17)	0.144
ECW/ICW ratio	0.71 ± 0.13	0.63 ± 0.14	−0.07 (−0.12, −0.03)	**0.003**	0.69 ± 0.14	0.61 ± 0.15	−0.08 (−0.16, −0.001)	0.050

*Note:* Differences reported are based on paired *t*‐test. *p*‐value < 0.05 (In bold).

Abbreviations: BCM, body cell mass; BFMI, body fat mass index; BMI, body mass index; ECW, extracellular water; FFMI, fat‐free mass index; LBM, lean body mass; PA, phase angle; SMMI, skeletal muscle mass index; TBW, total body water.

### 3.4 Trajectories of Body Composition During the Course of Cancer Therapy

**FIGURE 2:**
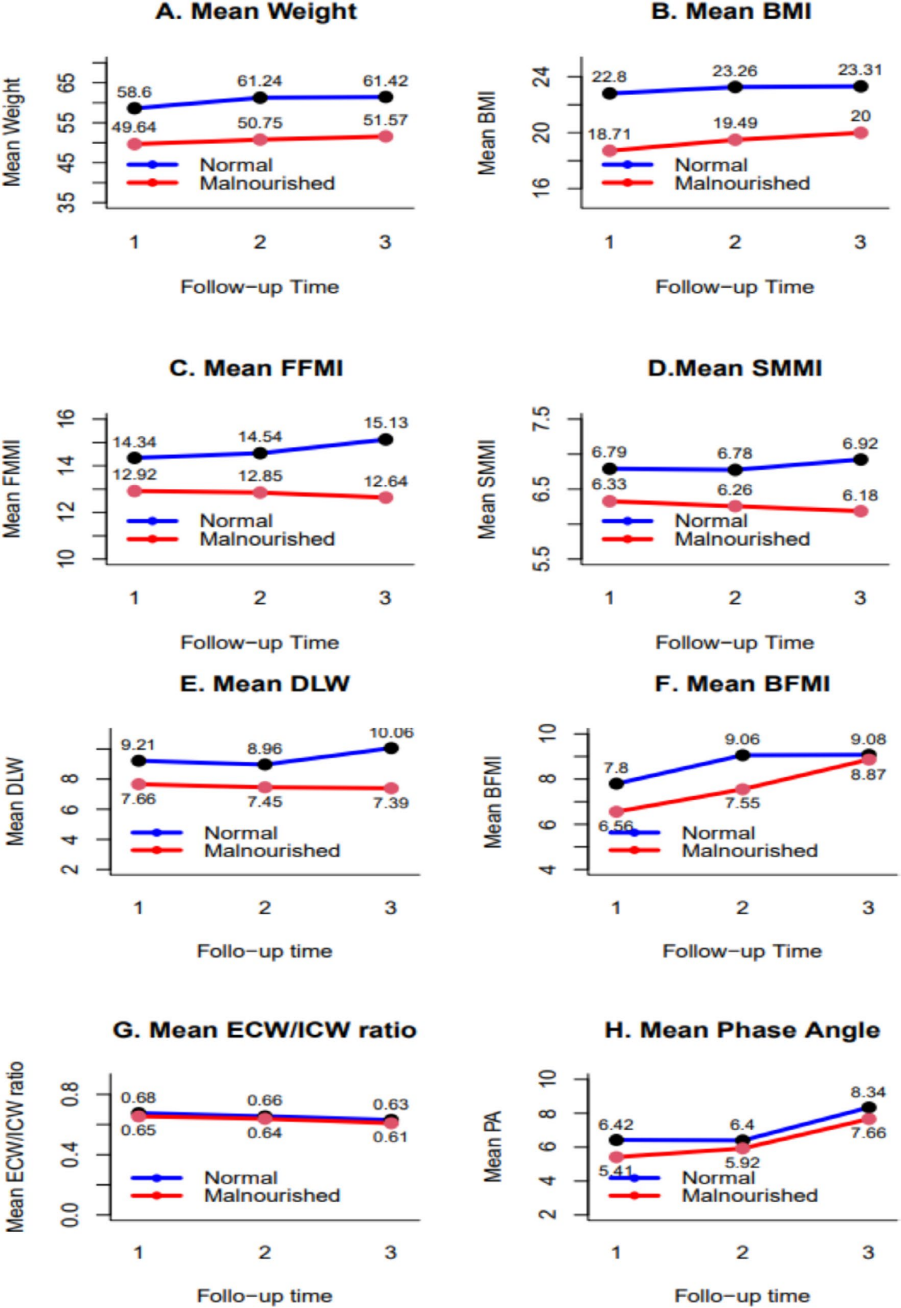
Mean change of body compositions overtime among newly diagnosed cancer patients during chemotherapy treatment at Jimma Medical Center, 2025. BMI, Body Mass Index; BFMI, Body Fat Mass Index; DLW, Dry Lean Weight; ECW, Extra Cellular Water; FFMI, Fat‐Free Mass Index; ICW, Intercw SMMI, Skeletal Muscle Mass Index; PA, Phase Angle.

Throughout the course of chemotherapy (baseline, 3 months, and 6 months), the mean values of body composition parameters including body weight, BMI, FFMI, SMMI, BFMI, and PA demonstrated linear increases among well‐nourished cancer patients. In contrast, among malnourished patients, only body weight, BMI, BFMI, and PA exhibited incremental changes. The study further revealed that the ECW/ICW ratio showed a consistent decline in both nutritional groups. Moreover, while mean values of DLW, SMMI, and FFMI progressively decreased in malnourished patients, these parameters increased in well‐nourished patients during the course of therapy (Figure 2).

### 3.5 Predictors of Variation of Body Composition Among Malnourished and Well‐Nourished Cancer Patients

This study demonstrated that age, sex, and cancer stage were significant predictors of changes in SMMI over time among newly diagnosed cancer patients with well‐nourished baseline status. In contrast, only age and sex predicted body composition change among malnourished patients. After adjustment for confounding variables and random effects, each one‐year increase in age was associated with a decrease in the mean evolution of SMMI by 0.033 (*β* = −0.033; 95% CI: −0.054, −0.013) in well‐nourished patients and by 0.042 (*β* = −0.042; 95% CI: −0.070, −0.015) in malnourished patients. Male sex was associated with an increase in the mean evolution of SMMI by 1.817 (*β* = 1.817; 95% CI: 1.264, 2.371) in well‐nourished patients and by 1.961 (*β* = 1.961; 95% CI: 1.190, 2.736) in malnourished patients. Furthermore, compared with stage I disease, stage II, III, and IV cancers were associated with decreases in the mean evolution of SMMI by 2.168 (*β* = −2.168; 95% CI: −3.658, −0.682), 2.260 (*β* = −2.260; 95% CI: −3.630, −0.866), and 2.491 (*β* = −2.491; 95% CI: −3.902, −1.082), respectively, among well‐nourished patients.

Similarly, age and cancer stage were predictors of the mean evolution of FFMI among well‐nourished patients, whereas sex alone was a predictor among malnourished patients. After adjustment, each one‐year increase in age was associated with a decrease in FFMI by 0.065 (*β* = −0.065; 95% CI: −0.118, −0.012) in well‐nourished patients. Compared with stage I disease, stage II, III, and IV cancers were associated with decreases in FFMI by 2.732 (*β* = −2.732; 95% CI: −4.631, −0.822), 2.286 (*β* = −2.286; 95% CI: −4.065, −0.500), and 2.511 (*β* = −2.511; 95% CI: −4.312, −0.691), respectively. Among malnourished patients, male sex was associated with an increase in FFMI by 0.713 (*β* = 0.713; 95% CI: 0.001, 1.420).

For BFMI, age, sex, and cancer stage were predictors among well‐nourished patients, whereas age and sex were predictors among malnourished patients. Each one‐year increase in age was associated with increases in BFMI of 0.064 (*β* = 0.064; 95% CI: 0.033, 0.096) and 0.082 (*β* = 0.082; 95% CI: 0.028, 0.136) in well‐nourished and malnourished patients, respectively. Male sex was associated with decreases in BFMI of 2.645 (*β* = −2.645; 95% CI: −3.484, −1.806) in well‐nourished patients and 1.125 (*β* = −1.125; 95% CI: −1.793, −0.457) in malnourished patients. Among well‐nourished patients, BFMI decreased by 2.966 (*β* = −2.966; 95% CI: −5.258, −0.675) in stage II and by 2.550 (*β* = −2.550; 95% CI: −4.694, −0.407) in stage III compared with stage I disease (Table 5).

**TABLE 5:** Predictors of body compositions among newly diagnosed cancer patients, Jimma Medical Center, 2025.

Body Composition parameters	Explanatory variables	Well‐nourished		Undernourished	
		*β* coefficient (95% CI)	*p*	*β* coefficient (95% CI)	*p*
SMMI	Age	−0.033 (−0.054, −0.013)	0.0022*	−0.042 (−0.070, −0.015)	0.0043*
	Sex(Male)	1.817 (1.264, 2.371)	< 0.001**	1.961 (1.190, 2.736)	< 0.001**
	Stage‐II	−2.168 (−3.658, −0.682)	0.0059*	1.066 (−1.259, 3.383)	0.3861
	Stage‐III	−2.260 (−3.630, −0.866)	0.0022*	1.591 (−0.639, 3.811)	0.1779
	Stage‐IV	−2.491 (−3.902, −1.082)	< 0.001**	1.373 (−0.774, 3.510)	0.2270
	Co‐morbidity	−0.069 (−0.846, 0.706)	0.8658	1.035 (−0.0.29, 2.101)	0.0678
	Duration of illness	0.001 (−0.021, 0.023)	0.9374	−0.003 (−0.019, 0.013)	0.6986
FFMI	Age	−0.065 (−0.118, −0.012)	0.0185*	0.012 (−0.041, 0.064)	0.6655
	Sex(Male)	0.563 (−0.139, 1.255)	0.1275	0.713 (0.001, 1.420)	0.0498*
	Stage‐II	−2.732 (−4.631, −0.822)	0.0076*	0.087 (−2.028, 2.203)	0.9385
	Stage‐III	−2.286 (−4.065, −0.500)	0.0167*	−0.648 (−2.685, 2.203)	0.5495
	Stage‐IV	−2.511 (−4.312, −0.691)	0.0097*	−0.448 (−2.397, 1.499)	0.6646
	Co‐morbidity	0.078 (−0.866, 1.026)	0.8763	−0.659 (−0.298, 1.614)	0.1957
	Duration of illness	0.015 (−0.013, 0.043)	0.3039	0.018 (−0.014, 0.051)	0.2800
BFMI	Age	0.064 (0.033, 0.096)	< 0.001**	0.082 (0.028, 0.136)	0.0040*
	Sex(Male)	−2.645 (−3.484, −1.806)	< 0.001**	−1.125 (−1.793, −0.457)	0.0014*
	Stage‐II	−2.966 (−5.258, −0.675)	0.0133*	0.011 (−1.951, 1.974)	0.9911
	Stage‐III	−2.550 (−4.694, −0.407)	0.0228*	−0.548 (−2.432, 1.336)	0.5774
	Stage‐IV	−2.097 (−4.278, 0.083)	0.0651	−0.890 (−2.689, 0.909)	0.3433
	Co‐morbidity	0.204 (−0.925, 1.332)	0.7283	0.851 (−0.042, 1.745)	0.0690
	Duration of illness	0.001 (−0.068, 0.08)	0.8170	−0.001 (−0.010, 0.008)	0.8050

*Note:* Estimates were derived from linear mixed‐effects models, which account for repeated measures within individuals. Missing measurements were retained in the dataset and handled under the model's assumption of missing at random (MAR), without removal or imputation.

Abbreviations: BFMI, body fat mass index; FFMI, fat‐free mass index; SMMI, skeletal muscle mass index.

* *p* < 0.05, but ≥ 0.001.

** *p* < 0.001.

## 4 Discussion

The ESPEN strongly recommends body composition analysis for newly diagnosed cancer patients [43] owing to monitoring body composition is vital for cancer survival, as its parameters can significantly impact outcomes [44]. However, resource‐limited settings like Ethiopia have yet to implement this practice. Therefore, this study used BIA to track patterns of body composition changes and identify their predictors during therapy in newly diagnosed cancer patients at the tertiary hospital of Ethiopia.

Accordingly, the findings of this study demonstrated that nearly all body composition parameters including body weight, BMI, LBM, DLW, SMMI, FFMI, BFMI, BCM, and PA were significantly lower in malnourished cancer patients compared with well‐nourished patients prior to initiation of cancer therapy. The low body composition in malnourished cancer patients before treatment highlights that baseline nutritional status is an important factor influencing change in body composition. Previous literature also testifies that under‐nutrition is associated with lower skeletal muscle mass, reduced BCM and altered FFMI [45]. Consequently, variations in body composition can impact treatment response, quality of life, overall survival and cancer‐specific survival. In particular, parameters such as low muscle mass, LBM and PA in cancer patients are associated with increased toxicity and poorer quality of life, overall and cancer‐specific survival [46, 47, 48]. Therefore, given its negative consequences, proactively assessing body composition and nutritional intervention as early as the cancer diagnosis is critical to optimal patient outcomes [20]. Similarly, this study indicates that malnourished cancer patients exhibited significant differences in body composition parameters compared with well‐nourished patients at the six‐month post‐therapy assessment.

The present study showed that notable LBM and ECW/ICW ratio decrement whereas BFMI and PA increment after chemotherapy in well‐nourished cancer patients. Nevertheless, among malnourished cancer patients, only BFMI showed a statistically significant increment after cancer therapy, while LBM and the ECW/ICW ratio exhibited numerical declines (Table 3). The reduction in LBM could imply that therapy may contribute to muscle wasting [46], and change in the ECW/ICW ratio may indicate shifts in hydration status or inflammation which could impact treatment tolerance and recovery [49, 50]. Meanwhile, the post‐therapy increase in body weight presents a paradox finding when considering changes in LBM. This discrepancy may be attributed to FM gain, which could mask components of LBM loss, such as BCM and the extracellular matrix [46, 51, 52].

Furthermore, our study has revealed that the decrement pattern of SMMI, FFMI, DLW, and ECW/ICW ratio among malnourished cancer patients, but an increment pattern was observed among well‐nourished individuals in almost all body composition parameters throughout the course of cancer therapy. This finding supported by finding conducted in Indonesia [18], Netherlands [46], South Korea [52], and China [53] implies that cancer patients with malnourished baseline nutritional status are at high risk of sarcopenia which can lead to increasing treatment toxicity and poor prognosis. Therefore, it is very important to assess baseline nutritional status and consider a multimodal individualized treatment plan to mitigate associated complications.

Additionally, the predictors of trajectories of body composition parameters in well‐nourished and malnourished cancer patients are identified in this study. SMMI decrement was noticed as age increased in both well‐nourished and malnourished cancer patients. The previous studies also support this finding [51, 54, 55]. A plausible explanation is that the co‐existence of aging, cancer, and cancer treatment like chemotherapy has a synergistic effect on skeletal muscle loss through various biochemical mechanisms [54]. However, in this study, the mean age of participants was 46.52 years, whereas the global median age at diagnosis for most solid tumors is approximately 65–67 years. This discrepancy warrants consideration of potential regional and demographic influences, including differences in population age structure, genetic predispositions, environmental exposures, and healthcare access patterns. Such factors may contribute to earlier cancer onset in certain populations [56, 57].

Being female is associated with a greater decline in SMMI as compared with males, potentially due to physiological differences such as hormonal influences [58]. The decrement pattern of SMMI was detected in advanced stages of cancer (stage IV and III) compared with stage‐I in this study, which is also supported by previous study finding [59]. This could be due to advanced cancer cells increasing metabolic rates, suppressing appetite, and reducing food intake by releasing systemic inflammatory factors such as TNF‐*α*, IL‐1, and IL‐6 [60]. This findings implies that late‐stage cancer identification significantly impacts patients' quality of life, and desired treatment outcomes through skeletal muscles mass loss [61]. Therefore, early‐stage cancer detection is essential to reduce complications associated with body composition changes and, in turn, enhance patients' quality of life.

Age and stage of cancer were identified as the predictors of FFMI and BFMI in the current study. As age increased by 1 year, the FFMI decreased by a factor of 0.065; this finding is supported by R. van Lieshout et al. [46], Bouh, et al. [51] and Jung, et al. [52]. This could be due to the loss of skeletal muscle with age increasing, as skeletal muscle is the component of FFM. In advanced cancer stages, both FFMI and BFMI show a decline. The potential justification for this finding could be in advanced cancer cells, the lipolysis and proteolysis commonly observed metabolic abnormalities [62, 63]. As age increased by 1 year, the BFMI increased by a factor of 0.064 and 0.082 in well‐nourished and malnourished cancer patients, respectively. Since most of our study population consists of menopausal females, the probability of fat accumulation increases in menopause age, which aligns with findings from a previous study [52].

This study has several limitations. First, the study population was heterogeneous with respect to cancer types and chemotherapy regimens. Such variability may constrain the interpretation of the findings, as differences in the diseases' characteristics and chemotherapy treatment regimens could influence outcomes. Second, the study experienced a substantial loss to follow‐up, which may have introduced bias. Therefore, the results should be interpreted with caution.

Despite these limitations, this study possesses several notable strengths. It evaluates the nutritional status of cancer patients using universally accepted, multidimensional criteria, incorporating BIA rather than relying solely on body weight. The prospective cohort design enhances the capacity to infer causal relationships between baseline nutritional status and trajectories of body composition during chemotherapy cycles. To minimize potential confounding, patients with a history of prior cancer therapy were excluded, thereby strengthening the internal validity of the findings. Moreover, longitudinal follow‐up at multiple time points enabled the assessment of the sustained impact of malnutrition on changes in body composition over time.

## 5 Conclusions and Implications for Research

At the baseline, newly diagnosed cancer patients exhibited significant differences in nearly all body composition parameters and body weight between well‐nourished and malnourished. This suggests that baseline nutritional status is a critical factor influencing variations in body composition and weight. During treatment, malnourished patients showed a decline in almost all body composition parameters except body weight, BFMI, though no statistically significant change was detected between pre and post‐treatment measurements. Therefore monitoring body weight alone may be misleading, as FM gain masks change in overall nutritional status. Prospective evaluation of body composition parameters such as SMMI and FFMI is crucial, as they offer more accurate and real patient's nutritional status than body weight alone. Therefore, we strongly recommend early nutritional assessment, incorporating body composition analysis at the time of cancer diagnosis and monitoring throughout the course of therapy, to mitigate potential complications. Additionally, this study highlights that advanced cancer stages, aging, and male sex were identified as key predictors of variations in body compositions across both nutritional groups.

This implies that late‐stage cancer identification significantly impacts patients' quality of life and desired treatment outcomes through skeletal muscle mass loss and diminished cell integrity or BCM. Therefore, early‐stage cancer detection is essential to reduce complications associated with body composition changes and in turn enhance patients' quality of life.

## Author Contributions

**Abebe Dukessa Dubiwak:** conceptualization, investigation, writing – original draft, visualization, writing – review and editing, validation, methodology, formal analysis, data curation. **Lelisa Sena Dadi:** data curation, supervision, formal analysis, methodology, validation, visualization, writing – original draft, funding acquisition, investigation, conceptualization, writing – review and editing. **Tefera Belachew:** conceptualization, writing – original draft, methodology, visualization, writing – review and editing, formal analysis, supervision. **Mulualem Tadesse:** investigation, conceptualization, funding acquisition, writing – original draft, writing – review and editing, formal analysis, project administration, supervision, data curation. **Henok Gulilat:** conceptualization, investigation, writing – original draft, funding acquisition, writing – review and editing, project administration, supervision. **Yohannes Tesfaye:** supervision, data curation, formal analysis, funding acquisition, investigation, conceptualization, writing – review and editing. **Belay Zawdie:** conceptualization, investigation, writing – original draft, visualization, writing – review and editing, project administration, supervision. **Selam Tesfaye:** conceptualization, investigation, funding acquisition, writing – original draft, methodology, visualization, writing – review and editing, project administration, supervision. **Melesa Sinaga Teshome:** conceptualization, investigation, funding acquisition, writing – original draft, writing – review and editing, visualization, validation, methodology, formal analysis, supervision, data curation.

## Funding

The authors have nothing to report.

## Ethics Statement

This study was conducted according to the guidelines laid down in the Declaration of Helsinki and all procedures involving patients were approved by the Research and Ethical Review Committee of the Institute of Health, Jimma University (Ref. No: JUIH/IRB/0432/24) on May 26, 2024. Verbal informed consent was obtained from all patients and it was witnessed and formally recorded.

## Consent

The authors have nothing to report.

## Conflicts of Interest

The authors declare no conflicts of interest.

## Data Availability

Data are available with the corresponding author based on reasonable request.
